# Phasing out the pre-transplant cytotoxicity crossmatch: Are we missing something?

**DOI:** 10.1590/2175-8239-JBN-2019-0222

**Published:** 2021-04-19

**Authors:** Jamile Abud, Bruna Brasil Dal Pupo, Cynthia da Silva, Elizete Keitel, Valter Duro Garcia, Roberto Ceratti Manfro, Jorge Neumann

**Affiliations:** 1 Santa Casa de Misericórdia de Porto Alegre, Laboratório de Imunologia de Transplantes, Porto Alegre, RS, Brasil.; 2 Universidade Federal do Rio Grande do Sul, Programa de Pós-Graduação em Medicina: Ciências Médicas, Porto Alegre, RS, Brasil.; 3 Santa Casa de Misericórdia de Porto Alegre, Centro de Nefrologia e Transplante Renal, Porto Alegre, RS, Brasil.

**Keywords:** Flow Cytometry, Cytotoxicity Tests, Immunologic, Graft Rejection, Transplante., Citometria de Fluxo, Testes Imunológicos de Citotoxicidade, Rejeição de Enxerto, Transplantation.

## Abstract

**Introduction:**

The anti-human globulin-enhanced complement-dependent cytotoxicity crossmatch (AHG-CDCXM) assay has been used to assess the presence of donor-specific antibodies (DSA) in recipient’s serum before kidney transplantation. The flow cytometric crossmatch (FCXM) assay was first introduced as an additional test. The aim of this study was to clinically validate the single use of the FCXM assay.

**Methods:**

This study compared the outcomes of a cohort of kidney transplant patients that underwent FCXM only (FCXM group) versus a cohort of kidney transplant patients that underwent AHG-CDCXM (control group).

**Results:**

Ninety-seven patients in the FCXM group and 98 controls were included. All crossmatches in the control group were negative. One patient in the FCXM group had a positive B cell crossmatch. One year after transplantation, there were no significant differences in patient survival (p = 0.591) and graft survival (p = 0.692) between the groups. Also, no significant difference was found in the incidence of Banff ≥ 1A acute cellular rejection episodes (p = 0.289). However, acute antibody-mediated rejections occurred in 3 controls (p = 0.028).

**Conclusion:**

The results showed that discontinuing the AHG-CDCXM assay does not modify the clinical outcomes in a 1-year follow-up.

## Resumo

**Introdução:**

O ensaio de prova cruzada por citotoxicidade dependente do complemento antiglobulina humana (AHG-CDCXM - do inglês anti-human globulin-enhanced complement-dependent cytotoxicity crossmatch) tem sido usado para avaliar a presença de anticorpos específicos contra o doador (DSA - do inglês donor-specific antibodies) no soro do receptor antes do transplante renal. O ensaio de prova cruzada por citometria de fluxo (CFXM) foi introduzido pela primeira vez como um teste adicional. O objetivo deste estudo foi validar clinicamente o uso único do ensaio CFXM.

**Métodos:**

Este estudo comparou os resultados de uma coorte de pacientes de transplante renal que foram submetidos apenas ao CFXM (grupo CFXM) contra uma coorte de pacientes de transplante renal submetidos ao AHG-CDCXM (grupo controle).

**Resultados:**

Foram incluídos noventa e sete pacientes no grupo CFXM e 98 controles. Todas as provas cruzadas no grupo controle foram negativas. Um paciente no grupo CFXM teve uma prova cruzada positiva para células B. Um ano após o transplante, não houve diferenças significativas na sobrevida do paciente (p = 0,591) e na sobrevida do enxerto (p = 0,692) entre os grupos. Também não foi encontrada diferença significativa na incidência de episódios de rejeição aguda celular (p = 0,289) segundo critério de Banff ≥ 1A. No entanto, rejeições agudas mediadas por anticorpos ocorreram em 3 controles (p = 0,028).

**Conclusão:**

Os resultados mostraram que a interrupção do ensaio AHG-CDCXM não modifica os desfechos clínicos em um acompanhamento de 1 ano.

## Introduction

Pre-transplant immunologic risk assessment is a key element in the clinical selection of potential recipients for a deceased donor kidney transplant. Sensitive and accurate tools for early detection of HLA antibodies in recipient serum, such as solid phase assays (SPA), allow the prediction of crossmatch results and help guide the use of immunosuppressive agents in the presence of donor-specific antibodies (DSA)^1^. Nonetheless, the B and T cell crossmatch remains essential to decision-making for transplantation in most centers^2^.

The complement-dependent cytotoxicity crossmatch (CDCXM) assay was proposed by Terasaki in 1969and has been commonly used to assess donor-recipient antibodies.^5^ Since then, modifications have been made to enhance its sensitivity, such as the addition of anti-human globulin (AHG), as some patients had no detectable antibodies on the CDCXM but suffered from acute antibody-mediated graft rejection and loss.^6^ A substantial increase in crossmatch sensitivity was observed with the use of the flow cytometric crossmatch (FCXM).^(7-9anti-HLA)^ Not only did the FCXM assay provide enhanced sensitivity but also required less time to be performed, leading to a reduction in cold ischemia time (CIT), which is inherent to deceased donor transplantation and one of the main predictors of initial graft function.^10^ In 2011, a new FCXM protocol was proposed by Liwski et al.^11^ The so-called Halifax protocol reduced even further the total assay time, thereby contributing to a significant decrease in CIT.

In this context, our laboratory adopted the Halifax FCXM protocol as the single pre-transplant crossmatch assay in September 2013. The present study assessed the clinical and laboratory outcomes in kidney transplant patients who underwent pre-transplant immunologic risk assessment with a single FCXM compared with patients from the period when CDCXM were used. The aim was to clinically validate the single use of the FCXM assay in the decision-making process for transplantation, and also to assess if the lack of information regarding complement fixing antibodies, the CDC cross match, could have any negative impact on our transplants.

## Patients and Methods

### Patients

This study was carried out at the Santa Casa de Misericórdia Hospital in Porto Alegre, in the Southern Brazilian state of Rio Grande do Sul. We followed a cohort of 100 kidney transplant patients who were selected consecutively and assessed with a single FCXM before transplantation (FCXM group). Similarly, we studied a retrospective cohort of 100 kidney transplant patients who were assessed with the CDCXM assays (control group). Five patients that received combined liver-kidney transplant were excluded. 

Adult and pediatric patients who received a kidney transplant from deceased donors from the state of Rio Grande do Sul, Brazil, were included in the study. The post-transplant follow-up period was 1 year.

### Immunologic risk assessment

The result of the panel-reactive antibody (PRA) tests performed in the patients’ sera in the last four months before transplantation was collected. Single-antigen bead (SAB) assays (LABScreen Single Antigen Beads, OneLambda, CA, USA) were performed in all recipients. The SAB protocol included heat treatment of the sera to minimize false-negative reactions. PRA scores for HLA class I and II antibodies were used, as well as specificity and mean fluorescence intensity (MFI) of HLA class I and II antibodies when these were present. The tests were conducted according to the manufacturer’s instructions, and the Luminex 100 system and the Fusion HLA software were used to analyze the results. The antibodies were considered positive if the MFI was higher than 1,000 and we considered DSA for HLA-A, -B and DRB1 for all patients. In patients typed for HLA-C and HLA-DQB1, these antibodies were also considered.

HLA typing of donors (HLA-A, -B, -C, -DRB1, -DQB1) and recipients (HLA-A, -B, -DRB1 in all and HLA-C and DQB1 in some) was performed by a sequence-specific primer set (SSP, OneLambda, CA, USA) according to the manufacturer’s instructions. The number of donor-recipient HLA mismatches were analyzed based on HLA typing for HLA-A, HLA-B, and HLA-DRB1.

Donor lymph nodes or spleen were used as sources of cells to perform the FCXM and CDCXM assays with the two latest recipient sera, stored at -80°C. Cells were separated by Ficoll-Hypaque density gradient centrifugation. The FCXM assay was conducted according to the Halifax protocol. Pronase treatment of lymphocytes was done,^12^ and T and B cells were assessed using peridinin-chlorophyll-protein complex (PERCP) anti-human CD3 (clone SK7, BD Biosciences) and phycoerythrin (PE) anti-human CD19 (clone HIB19, BD Biosciences). Fluorescein isothiocyanate (FITC) F(ab’)2 Anti-Human IgG, Fc fragment specific (Jackson ImmunoResearch Laboratories, USA) was added. The samples were collected and analyzed with the BD FACSCalibur flow cytometer (BD Biosciences), and cut-off scores were set at 40 for T cells and 100 for B cells. The samples for CDCXM were treated with dithiothreitol, and anti-human globulin (AHG-CDCXM) assay was performed for T-cells and CDCXM not modified was performed for B cells. The protocols were conducted according to the American Society for Histocompatibility and Immunogenetics (ASHI) protocol^13^ using a fluorescent marker for dead cell quantification and magnetic beads for T and B cell separation. 

### Clinical and predictive variables

Demographic data of donors and recipients were collected. Donors were classified as expanded criteria donors (ECD) according to the definition of the United Network for Organ Sharing (UNOS). Data on CIT, underlying diseases, and previous transplantation were collected from the recipients’ electronic medical records. In the study period, there were no changes in the immunosuppression protocols of the transplantation center. All DSA-negative transplant patients were treated with the anti-CD25 monoclonal antibody (interleukin-2 receptor). Patients with PRA score higher than 50%, DSA-positive patients, and patients whose donors had CIT higher than 24 hours were treated with anti-thymocyte globulin (ATG). The maintenance therapy consisted of tacrolimus, mycophenolate, and prednisone.

### Clinical outcomes

Protein-to-creatinine ratio (PCR) and estimated glomerular filtration rate (eGFR) were evaluated at 3, 6, and 12 months after transplantation. eGFR was calculated by the Modification of Diet in Renal Disease (MDRD) Study equation^1^ in adult patients and by the Schwartz equation in patients younger than 18 years In patients who underwent post-transplant SAB testing, the presence of de novo DSA was determined. Delayed graft function (DGF) was defined as the need for dialysis until the seventh day after transplantation, and DGF length was measured. Rejections were categorized based on the interpretation of the transplant pathologist according to the Banff 2007 classification.^17^ Acute antibody-mediated rejection (ABMR) was assessed according to the Banff 2013 classification. Graft loss was defined as the need to resume dialysis, and the causes of graft loss were collected from medical records. Deaths and their causes were collected from medical records and reviewed by a physician from the kidney transplantation team.

### Statistical analysis

StatCalc and SPSS, version 20, were used for the statistical analyses. Categorical variables were expressed as absolute frequencies (number of patients) and relative frequencies (percentage). Parametric data were compared using the Student’s t-test, while non-parametric data were compared using the Mann-Whitney U test. Categorical variables were compared using the chi-square test. The Kaplan-Meier survival analysis was used for patients and grafts. Multivariate analyses not were done. For statistical purposes, a significance level below 0.05 was set. The Strengthening the Reporting of Observational Studies in Epidemiology (STROBE) checklist was used to guide the study.

### Ethical aspects

This study was approved by the Research Ethics Committee of Santa Casa Hospital from Porto Alegre, state of Rio Grande do Sul, southern Brazil, under protocol number 41095914.1.0000.5335.

## Results

### Donor and recipient characteristics at the time of transplant

We assessed 100 patients in the FCXM group, but 3 were excluded (kidney transplants performed between October 2013 and October 2014) and 100 patients in the control group (kidney transplants performed between October 2012 and September 2013). Five patients were excluded because of combined liver-kidney transplants. Pre-transplant demographic and clinical data of the groups are shown in Table 1.

**Table 1:** Baseline characteristics of kidney transplants in the studied groups

Variables	Total number of patients	FCXM group (n=97)	Control group (n=98)	p
**Donors**	195			
Male – n (%)		46 (47.4)	70 (71.4)	<0,001
Female – n (%)		51 (52.6)	28 (28.6)	
Age - years; mean (SD)	195	41 (18.8)	40 (21.4)	0.124
**Age - years; mean (SD)**	195	41 (18.8)	40 (21.4)	0.124
**Mismatch HLA- A, HLA-B, HLA-DR- mean (SD)**	195	4 (1.2)	4 (1.2)	0.542
Recipients				
Male – n (%)	195	60 (61.9)	52 (53.1)	0.273
Female – n (%)		37 (38.1)	46 (46.9)	
Age at transplant – years; mean (SD)	195	44 (18.3)	45 (19.7)	0.653
Primary kidney disease – n (%)	195			0.09
Unknown		46 (47.4)	35 (35.7)	
Hypertension		10 (10.3)	26 (26.5)	
Diabetes		15 (15.5)	15 (15.3)	
Polycystic kidney disease		9 (9.3)	5 (5.1)	
Others		17 (17.5)	17 (17.3)	
Number of kidney transplants – n (%)	195			0.833
First		80 (82.5)	79 (80.7)	
Second or third		17 (17.5)	19 (19.3)	
Class I DSA – n (%)	195			0.803
Absent		83 (85.6)	83 (84.7)	
1		9 (9.3)	12 (12.2)	
2		4 (4.1)	3 (3.1)	
3		1 (1.0)	-	
Class II DSA – n (%)	195			0.277
Absent		84 (86.6)	92 (93.9)	
1		9 (9.3)	5 (5.1)	
2		3 (3.1)	1 (1.0)	
3		1 (1.0)	-	
DSA MFI-Sum – median (IQR)				
Class I (FCXM group n=14; control group n=15)	29	2,342 (1,203-3,408)	2,669 (1,794-3,845)	0.270
**Class II (FCXM group n=13; control group n=06)**	19	3,359 (1,462-8,436)	1,838 (1,212-6,553)	0.467
**ECD -** n (%)	77	38 (40%)	39(39,8)	0.977
**CIT – time in hours; mean (SD)**	185	20.4 (5.5)	20.3 (4.9)	0.342

DSA MFI-Sum: sum of the mean fluorescence intensity of the different circulating donor-specific antibodies; FCXM: flow cytometric crossmatch.

Deceased donors were all brain dead. There were 116 male donors overall, including 46 in the FCXM group (47.4%) and 70 in the control group (71.4%) (p < 0.001). Mean donor age was 41±18.8 years in the FCXM group and 40±21.4 years in the control group (p = 0.124). Thirty-eight donors in the FCXM group (40%) and 39 controls (39.8%) were classified as ECD, with no significant between-group difference (p = 0.977). CIT did not differ between the groups (FCXM group: 20.4±5.5 hours; control group: 20.3±4.9 hours; p = 0.342). HLA-A, -B, and -DRB1 types were available for all donors, while HLA-C and -DQB1 types were typed in 60.5% (n = 118) and 63.5% (n = 124) of the donors, respectively. No donor was genotyped for HLA-DPB1.

There were no significant differences between mean PRA scores for anti-HLA class I antibodies (FCXM group: 21.0%±31.0; control group: 17.2%±29.3; p = 0.427) and anti-HLA class II antibodies (FCXM group: 13.9%±22.6; control group: 15.1%±24.4; p = 0.315). DSA, either class I or II, were absent in 86% of the patients before transplantation. Pre-transplant PRA scores are shown in Table 1. The mean number of mismatches for the HLA-A, -B, and -DRB1 loci was 4 ±1 (p = 0.542) in both groups. 

### Clinical and immunological outcomes

One hundred and fifteen recipients (59%) presented DGF, with no significant difference between the groups (Table 2). Estimated GFR and urinary protein excretion (total protein/creatinine in a urine sample) were assessed at 3, 6, and 12 months after transplantation. Urinary PCRs were available from 128 patients at 3 and 6 months post-transplant and 127 patients at 12 months post-transplant. eGFR analysis was made separately for patients aged less than 18 years and patients aged 18 years or older. No differences in eGFR were observed over time in the group of recipients younger than 18 years. In adult recipients, a significantly higher eGFR was observed in the FCXM group at 12 months after transplantation (Table 2).

**Table 2:** Graft function outcomes

Variables	Number of patients evaluated	FCXM group	Control group	p
DGF – n (%)	195	59 (60.8)	56 (57.7)	0.770
DGF, days – mean (SD)		5.5 (4.0)	4.58 (3.5)	0.280
PCR, median (IQR)				
3 months	128	0.30 (0.2-0.5)	0.37 (0.2-0.5)	0.120
6 months	128	0.28 (0.2-0.5)	0.36 (0.2-0.7)	0.240
12 months	127	0.23 (0.2-0.5)	0.33 (0.2-0.6)	0.150
eGFR < 18 years (mL/min/1.73m^2^), median (IQR)				
3 months	21	65 (53-72)	70 (65-75)	0.223
6 months	21	65 (57-68)	65 (60-75)	0.605
12 months	20	81 (54-103)	65 (55-71)	0.412
eGFR > 18 years (mL/min/1.73m^2^), median (IQR)				
3 months	157	38.0 (29.5-49.0)	37.5 (17.0-51.0)	0.267
6 months	146	43.5 (29.0-54.3)	40.0 (15.0-50.8)	0.090
12 months	128	46.0 (33.0-57.0)	39.0 (22.5-49.0)	0.009

DGF: delayed graft function; eGFR: estimated glomerular filtration rate; FCXM: flow cytometric crossmatch; PCR: protein-to-creatinine ratio.

All T and B cell crossmatches were negative in the control group. In the FCXM group, one patient presented a positive B cell FCXM with a 193 channel shift. The positive finding was attributed to an anti-HLA-DQ6 DSA. The recipient underwent a prior kidney transplant, had Banff type IB acute cellular rejection, and maintained a functioning graft at the end of the follow-up period.

Sixteen patients from the FCXM group underwent a clinically indicated SAB test in a mean post-transplant time of 82.0±22.9 days, while 18 controls underwent the same test in a mean post-transplant time of 87.0±19.5 days (p = 0.852). In the FCXM group, 4 patients had class I DSA and 3 had class II DSA, while in the control group, 7 patients had class I DSA and 4 had class II DSA (Table 3).

**Tabela 3:** Presence of DSA after transplantation

DSA	FCXM group(n = 16)	Control group(n = 18)	p
**Class I – n (%)**			0.558
Absent	12 (75)	10 (55.6)	
1	2 (12.5)	3 (16.7)	
2	1 (6.3)	1 (5.6)	
3	1 (6.3)	4 (22.2)	
**Class II – n (%)**			0.942
Absent	13 (81.3)	14 (77.8)	
1	2 (12.5)	3 (16.7)	
2	1 (6.3)	1 (5.6)	
De novo DSA – n (%)	5 (5.2)	8 (8.2)	0.400

DSA: donor-specific antibodies; FCXM: flow cytometric crossmatch.

### Survival analysis

**Figure 1:**
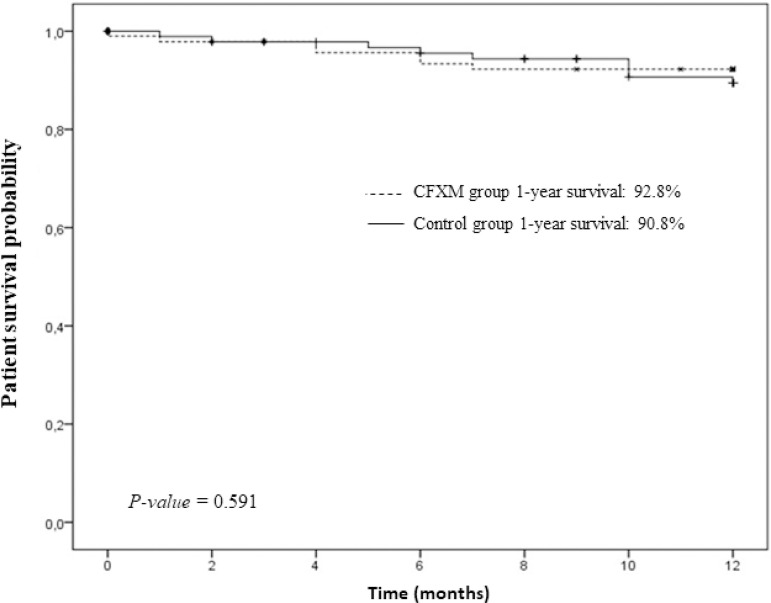
Patient survival rate.

**Figure 2:**
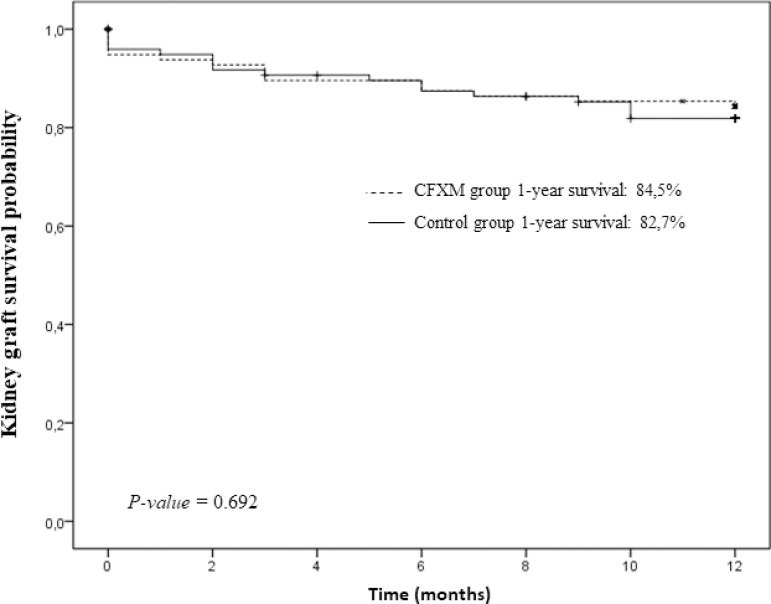
Graft survival rate.

One year after transplantation, there were no significant differences in patient survival (FCXM group: 92.8%; control group: 90.8%; p = 0.591) and graft survival (FCXM group: 84.5%; control group: 82.7%; p = 0.692) (Figures 1 and 2). Sixteen patients died in the follow-up period, 7 in the FCXM group and 9 in the control group (p = 0.811), most of them (n = 11) due to infections. There were 15 (15.5%) graft losses in the FCXM group and 17 (17.3%) in the control group, with no significant between-group difference (p = 0.872). Two failures occurred due to antibody-mediated rejection in the control group, while no graft loss due to immunological causes occurred in the FCXM group.

### Graft biopsy data

Sixty-three (48%) patients in the FCXM group and 68 (52%) controls underwent a kidney graft biopsy. As shown in Table 4, no significant difference was found in the incidence of acute cellular rejection equal to or greater than 1A [16] (p = 0.289). However, acute ABMR occurred in 3 patients in the control group and none in the FCXM group (p = 0.04). In the 3 patients with ABMR, none had pre-transplant DSA, all received grafts from ECD donors, had DGF and formed de novo DSAs, two lost the graft, and none died. C4d deposits along peritubular capillaries were absent in 47 (85.5%) and 30 (49.2%) patients in the FCXM group and the control group, respectively. Any level of C4d intensity was detected in 8 (14.5%) and 31 (50.8%) patients in the FCXM group and the control group, respectively (p < 0.001).

**Table 4:** Histopathologic findings in graft biopsy

Pathology test results – n (%)	FCXM group	Grupo controle (n = 68)	p
(n = 63)	Control group	20 (29.4)	0,750
(n = 68)	p	13 (19.1)	0,638
No rejection	16 (25.4)	20 (29.4)	0.750
Borderline rejection	10 (15.9)	13 (19.1)	0.638
Acute cellular rejection ≥ 1A	27 (42.9)	21 (30.8)	0.293
Acute antibody-mediated rejection	-	3 (4.5)	0.028
Interstitial fibrosis and tubular atrophy	1 (1.6)	5 (7.4)	0.246
Other findings	9 (14.3)	6 (8.8)	0.667

CFXM: prova cruzada por citometria de fluxo.

## Discussion

In the present study we assessed the FCXM as the only crossmatch assay in patients undergoing a deceased donor kidney transplant. No significant differences were found in the main clinical outcomes of the group that underwent FCXM alone compared with the group that underwent AHG-CDCXM (controls).

There were no statistically significant differences in the incidence of DGF and urinary PCR one year after transplantation. eGFR was higher in the FCXM group than in the control group. Among those recipients who underwent clinically indicated SAB testing, most patients from both groups did not develop DSA one year after transplantation. Importantly, patient and graft survivals were not significantly different between the groups. The incidence of acute cellular rejection was not different between groups. However, three cases of acute ABMR were observed in the control group compared with none in the FCXM group.

The studied groups were homogeneous in terms of risk predictors (donor age, underlying disease, CIT, number of HLA mismatches, pre-transplant PRA score, and DSA screening), which contributed to reducing biases in the analyses. In order to reduce variability in organ quality, transplants of organs coming from other Brazilian states were not included. In both groups, the presence of DSA was evaluated through SAB assay before transplantation.

In a 2008 study of 354 kidney transplant patients, Ho et al.^19^ solid phase (SPA) evaluated the sensitivity and specificity of the CDCXM, FCXM, and SPA assays using graft loss as the main outcome. These three tests were performed in all patients to assess the presence of DSA. There was no significant difference in graft survival between these methods in a 3-year follow-up for both first transplant and re-transplant patients. The authors reported the importance of the CDCXM and FCXM assays according to each method’s sensitivity. Their results are consistent with our findings, although the two studies were designed differently, as the FCXM assay was used as the single crossmatch method in our study. In consonance with our findings, a former retrospective U.S. study examined survival and clinical outcomes in 624 kidney transplant patients, mostly from deceased donors, tested only with the FCXM assay and divided into three groups (T^-^ B^-^ FCXM, T^-^ B^+^ FXCM, and T^+^ B^+^ FXCM), and reported a 1-year graft survival of 90% in the T^-^ B^-^ FCXM group.^20^^-^ 22

Unlike the AHG-CDCXM assay, the FCXM assay stratifies the risk and might not necessarily contraindicate transplantation when the result is positive. Graff et al. (2009) studied retrospectively the outcome implications of positive FCXM results, using data for a national cohort of transplant recipients recorded in the Organ Procurement and Transplant Network registry data. They observed a continued detrimental effect of a positive FCXM result beyond the first transplant anniversary.^23^ We had one recipient transplanted after B^+^ FCXM in the control group and this patient was free of dialysis three years after transplantation.

Our laboratory used to perform both the AHG-CDCXM and FCXM assays by the standard ASHI protocol. The Halifax protocol encouraged us to adopt the FCXM assays as the sole cross matching evaluation. This strategy allowed a reduction in the time required to perform the test. Similarly, de Moraes et al.^24^ concluded in their study that the exclusive use of FCXM as a cell test for pre-transplantation evaluation of anti-donor antibodies is feasible given the safety in terms of predicting CDC negative results and by assessing the risk of a preformed DSA. However, contrary to our expectation, the CIT did not decrease. This can be explained by the fact that the process of organ donation involves multiple teams and factors that are independent of the crossmatch assay.

This study had some limitations. Firstly, it was a single-center, non-randomized study with a retrospective control group. We believe that this limitation did not impact the results as the overall medical practice, including immunosuppressive regimens, was essentially the same throughout the study period. Secondly, post-transplant DSA results were not available for all recipients, and HLA-C and DQB1 loci were not typed for all recipients. However, the number of recipients with post-transplant SAB testing was similar between groups suggesting a similar clinical need for such testing in clinical practice. Finally, we did not perform a formal cost-benefit analysis, comparing the two techniques. 

## Conclusions

The sensitivity of the methods used to detect HLA class I and II antibodies have constantly been increased as a result of advances in tests such as the FCXM and SPA assays. The main purpose of the present study was to demonstrate that discontinuing the use of the AHG-CDCXM assay does not modify the clinical outcome of kidney transplants. A combined assessment using the SAB test and the FCXM assay should be performed to evaluate risks and help the decision-making process Even though the higher sensitivity of the FCXM is well recognized, this method is seldom used alone outside North America. Therefore, we believe that validating its clinical application by reporting our experience could be a contribution to the field. Also important, the FCXM assay is far from standardized. Only recently a proposed standard protocol, the Halifax protocol, was published.^12^ Finally, it is important that centers validate their own FCXM results with respect to acceptable clinical risks.We are confident that the results described here strongly support the safety of using the Halifax FCXM as the only pre-transplant crossmatch test. Importantly, the lack of information regarding complement-fixing antibodies, as in the CDCXM assays, does not have a detrimental impact on the quality of kidney transplantation in our practice.
